# Supplementary material for: Induction of Invasive Basal Phenotype in Triple-Negative Breast Cancers by Long Noncoding RNA BORG
Source: Cancers (Basel). 2024 Sep 23;16(18):3241. doi: 10.3390/cancers16183241 (PMC11430157; doi:10.3390/cancers16183241)
Supplement: Supplementary file 1 [file cancers-16-03241-s001.zip › cancers-3092769-supplementary.pdf]

Supplementary Figures

The putative polyadenylation signal of human BORG RNA

Genomic DNA hg38 chr8:103095055-103095458 strand=+ repeatMasking=none

```
ATGATAGATATAATTATATGAAAAAGTTTGAAATATTGTGAGAATTACCA
AAATGTGACACAGAGACGTGAAGTAAGCACGTGCTGTTGGAAAATGGACT
T/GATCAATGCAGGGTTTTCACACACCCCCAATTTATTAAAATCACAATAT
TTGCACAGCACAATAAAATGAAGTACAATAAAATGGGGTATGTTTATACA
TAAGAGATCAAATTAATAACTTCCAACTGCAACAGCCATAGGCCACCAAT
GATGTTGAAGTTATTAACATATAGAGTGTAAGGAATTGAACTTTGATTCT
TACATAATTCCTGCAACAGACCTAATGGTAATGAACTGGGGGAAATCCAG
CAAGCCCTGTTCAGATTCATTTCTGTGTCCCCTTGTCTTCAGGGATGAGA
ACGT
```

hg38 chr 8: 103,095,186: the putative polyadenylation signal AAUAAA sequence (here read as TTTATT in antisense, highlighted in yellow)

hg38 chr 8: 103,095,156: the putative cleavage site (highlighted in purple, C/A, here in antisense read as T/G, 30 nts downstream of the putative polyA signal).

Fig. S1. BORG is transcribed in the antisense standard genomic direction. Position of the canonical poly(A) signal (AAUAAA hexamer, here in antisense direction, highlighted in yellow) at the 3' end of BORG is shown. The putative cleavage site (highlighted in purple) which is located 30 nucleotides downstream of the AAUAAA hexamer is also marked. The genomic coordinates of the poly(A) signal hexamer and the cleavage site is listed at the bottom of the figure.

hg38 chr8: 103,109,058-103,109,425

```
human           AGCAGGAGCGATGGGTCACATGACAAAGAGAGAAGCAGGTTTTCTCTCCACATCTGATAA
chimp           AGCAGGAGCGATGGGTCACATGACAAAGAGAGAAGCAGGTTTTCTCTCCACATCTGATAA
macaca          AGCAGGATCGATAGGTCACATGACAAAGAGAGAAGCAGGTATTCTCTCCACATCTGATAA
cow             -------------------------------------GGTTTTCTCTCTACATTAAATAA
pig             ---------GATGGCTCAGGTGACCTAGAGAGAAGCGGCTTTTCTCTCTACCTGAAGTCA
cat             ------------------------------------------------------------
dog             ------------------------------------------------------------
mouse           AGCGGGAACCAGGGCTCCTATGACAGAAA-AGAAGCAGGC-TTCTCTCTGCAATTCATAA
rat             AGCAGGAACCAGAGCTCTTCTGACAGAAA-AGAAACAGGC-TTCTCTCTGCGTTTCATAG

human           ACACTGACAAGAGTTAGGTGATTTTCCTACACACAGCACTATCTTGTTCTGAGAAAGGAT
chimp           ACACTGACAAGAGTTAGGTGATTTTCCTACACACAGCACTATCTTGTTCTGAGAAAGGAT
macaca          ACACCGACAAGAGTTAGGTGATTCTCCTACACATGGCACTATCTTGTTCTGAGAAAGGAT
cow             ACACTAACAAGAGTTAGGTGATCCCCCT-CACCCTACACTTCCTTGTTCAGAGAAAGGAT
pig             ATGCTGACAAGAGTTGGGTGATCTTCCTACACCCACCGCTGTCTGGTTCAGAGGAAGGGT
cat             ------------------------------------------------------------
dog             -------------------------------------------TTGTTCTGAGGAAGGAT
mouse           ACACCGACAGGAATATGTTGCTTTTCCTAAGCCCTTCGCTGTCTTTCCAAGAAGAATTTT
rat             ACACTGACAAGAGTCGGATGCTTTTCCTAGGCCCTGCGCTGCCTTTCCAAGAAGAATCTT

human           TTG--CCTTAACCAAATACGGGGCCTGACAGCTTTTTCTCTCAAATGAATCAGTGGGGAG
chimp           TTG--CCTTAACCAAATACGGGGCCTGACAGCTTTTTCTCTCAAATGAATCAGTGGGGAG
macaca          TTG--CCTCAACCAAATACAGGGCCTGACAGCTTTT-CTCTCAAATGAATCAGTGGGGAG
cow             CTG--ACTTCACCAGACATAAAACCTGACAGGTATTTCTCGCGAATGAATCAGTGGGGAG
pig             TTG--ACTTTACCAAATAGAAAACCTGACAGGTAGGCCTCCCCAGTGAATCAATGGGGTG
cat             -----------------------------------TTCTCCCAAATGAGTCAGTGGGGAG
dog             CTG--ACTCAACCA-ATACAGAACCTGACA--TATCTCTCTCAAATGAATCAGTGGGGAG
mouse           TTTGTCCTTAACCAAATGTGAAAT---GCACATTTTGAGCACAAA-GAATCAAAGCTCCA
rat             TTCATCTTTAACCAAATAGGAA------CTGCTGGTGCGCATAAATGAATGAATGCTTCA
                                                       *   *  ** * *  *
human           GAGCTGAAGTGACTTTTTGTTTTG---AAAGAAATGCAATATTTAAAGAGAGA------G
chimp           GAGCTGAAGTGACTTTTTGTTTTG---AAAGAAATGCAATATTTAAAGAGAGA------G
macaca          GAGCTGAAGTGACTTTTTGTTTTG---AAAGACATGAAATATTTAAAGAGAGA------G
cow             GACCTGAAGCGACTTAGTTTTTTATCTAAAGAAATGTAATATTTAAAGAAAGA------G
pig             GAGCTGAAGTGACTTTTTTTTAAATTTAAAGAAATGTAATATTTAAAGA----------G
cat             GAGCTGAAATGGCAGTTGTTTAAGA--AAAGGAGTGTAATATTTAAAGAGAGT------T
dog             GAGCTCAAATGACATTTGGGGGGGG--AAAGAAATGTAATATTTAAAGAGA--------G
mouse           ACAGCAAACTGACATGACATCCTTTA-CAGGAAGTAAAATATTTAAAGACAGAGTCAGAG
rat             ACAGCAGGCTGATGCGACATCCTTTA-AAGGAAGAGAAATATTTAAAGACAGA------G
                          *                 * *      ************


human           GTTTCTATTTTTCCCC-CA-GGAGAAGGGAGCCCTTAATATTCCCATCAACGATGACACA
chimp           GTTTCTATTTTTCCCC-CA-GGAGAAGGGAGCCCTTAATATTCCCATCAACGATGACACA
macaca          TTTTCTATTTTTCCCC-TA-GGAGAAGGGAGCCCTTAATATTCCCATCAACGATGACACG
cow             TTCGCTATCCCCCCTC-TTTGGGGAAGGGAGCCCTTAATTTTCCCATCAACTCTGACACA
pig             TTTTCTATTCCCCCTC-CTTGGGGAAGAGAGCCCTTAATTTTCCCATCAACTCTGACATG
cat             TTCTGCTTCCCCCTCG-CTTGGGGAAAGGAGCCCTTAATATTCCCATCAACCGTGACACG
dog             TTCTCAGTCCCCCCTCGCTTGGGGAAGGGAGCCCTTAATCTTCCCATCAAC---------
mouse           TCTTCTATTCCCCTCC-CCCCAGGAAGAAACCCTTTAATATTCCCATTAACCTTGA----
rat             TCTTCTATTTTCCCCC---TGAGGAAGAAACCCTGTAATATTCCTATTAACCTTGACATG
                       *    *          ***   * **  **** **** ** ***
```

```
human           TGTGAATCACAGCCCTTTCTT-TCCTATAAAAGCA-----TCATGATTCAACACAAAGCC
chimp           TGTGAATCACAGCCCTTTCTT-TCCTATAAAAGCA-----TCATGATTCAACACAAAGCC
macaca          TATGAATCACAGCCCTTTCTT-TCCTATAAAAGCA-----TCATGATTCATTACAAAGCC
cow             TGTGAATCACAGCACTTTCAG-TCCTATAAAAGCC-----TCATGATTCATCAACAACCC
pig             TGTGAATCACAGCCCTTCTGT-TCCTATAAAAGCA-----TCATGATTCATCAGCAGCCT
cat             TGTGAATCACACCCCTTTCGC-TCTTACAGAAGCG-----GCATGATTCATCGCGAACCC
dog             ------------------------------------------------------------
mouse           TGTGAATCACAGCGCTCCTTC-TTCCCTGTAGGCAGATAATCATGACTCACCACAGACCC
rat             TGTGAATCACTGAGCTCTTTCATCCCCTGTAGGTAGATTATCATGACTCACCACAGACCC

human           CGCC----AGCTGTGGGCTGCATGTGATTCT----
chimp           CGCC----AGCTGTGGGCTGCATGTGATTCT----
macaca          CGCC----AGCTGTGGGCTGCATGTGATTCT----
cow             TTCT----AGCTGTGGGCTGC--------------
pig             TGCC----AGCTGTGGGCTGCA-------------
cat             TGCCCGCCAGCTGTGGGTTGCGTGTTATTCT----
dog             -----------------------------------
mouse           CT------GACTGTGGGCTTCATGTAATTCTGGCA
rat             AC------GACTGTGGGCTTCATGTAACCCT----
```

Fig. S2. Alignment of the phylogenetically conserved region of BORG located close to the 3’ end of the main BORG transcript isoform in the mouse among nine mammalian species. This region is shown in Fig. 1A as the rightmost red line above the BORG gene model.

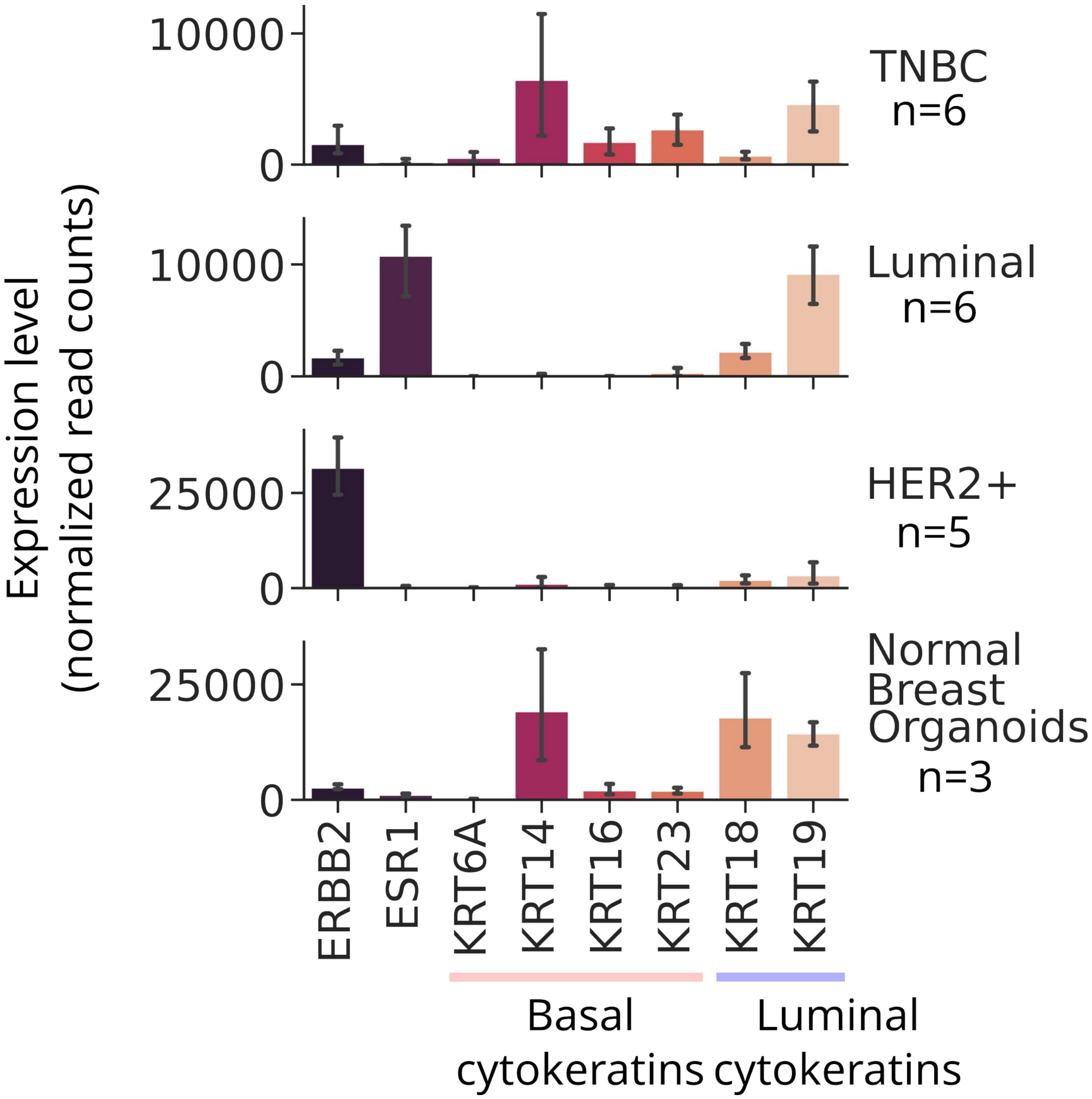

Fig. S3. Validation of the HER2+, luminal and triple negative phenotype in the breast cancer tumors analyzed in Fig. 1B. RNA-level expression of HER2 (ERBB2), ER (ESR1) and basal and luminal-specific cytokeratins are shown. Number of tumors in each category is shown to the right.

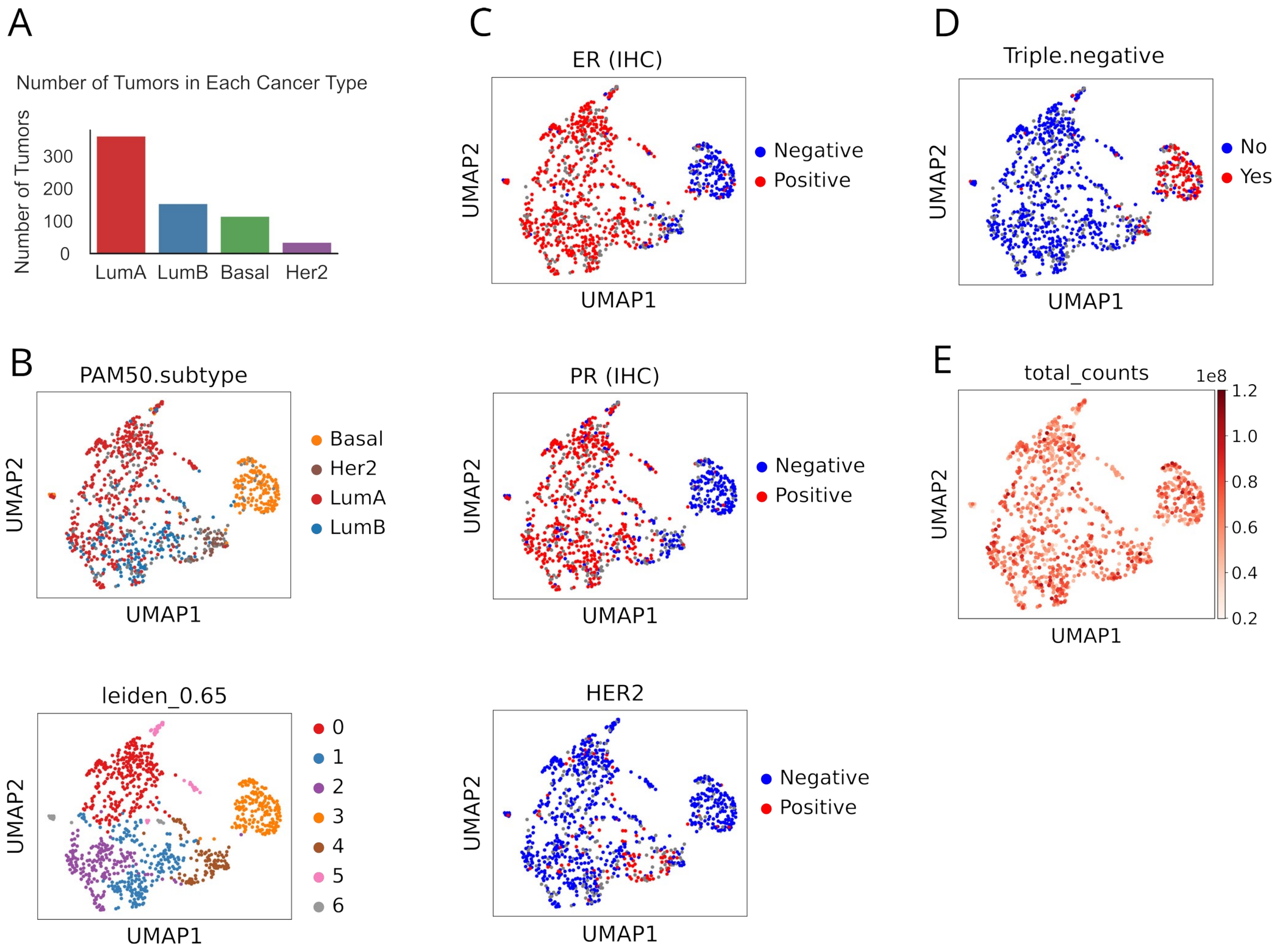

Fig. S4. The TCGA BRCA dataset samples used in this study, after the filtering steps described in Methods. A. Number of tumors in each PAM50 histological subtype after filtering. B. UMAP representation of the TCGA BRCA cohort samples used in this study. PAM50 subtypes closely follow the pattern of unbiased leiden clustering, indicating distinct transcriptional patterns among BC subtypes. Tumors removed during the filtering process are shown in gray. C. Expression pattern of ER, PR and HER2 at protein level based on the annotations provided by TCGA. D. TNBC tumors are segregated in a separated cluster compared to Luminal A, B and HER2+ tumors. E. Depth of sequencing in TCGA BRCA tumors used in this study.

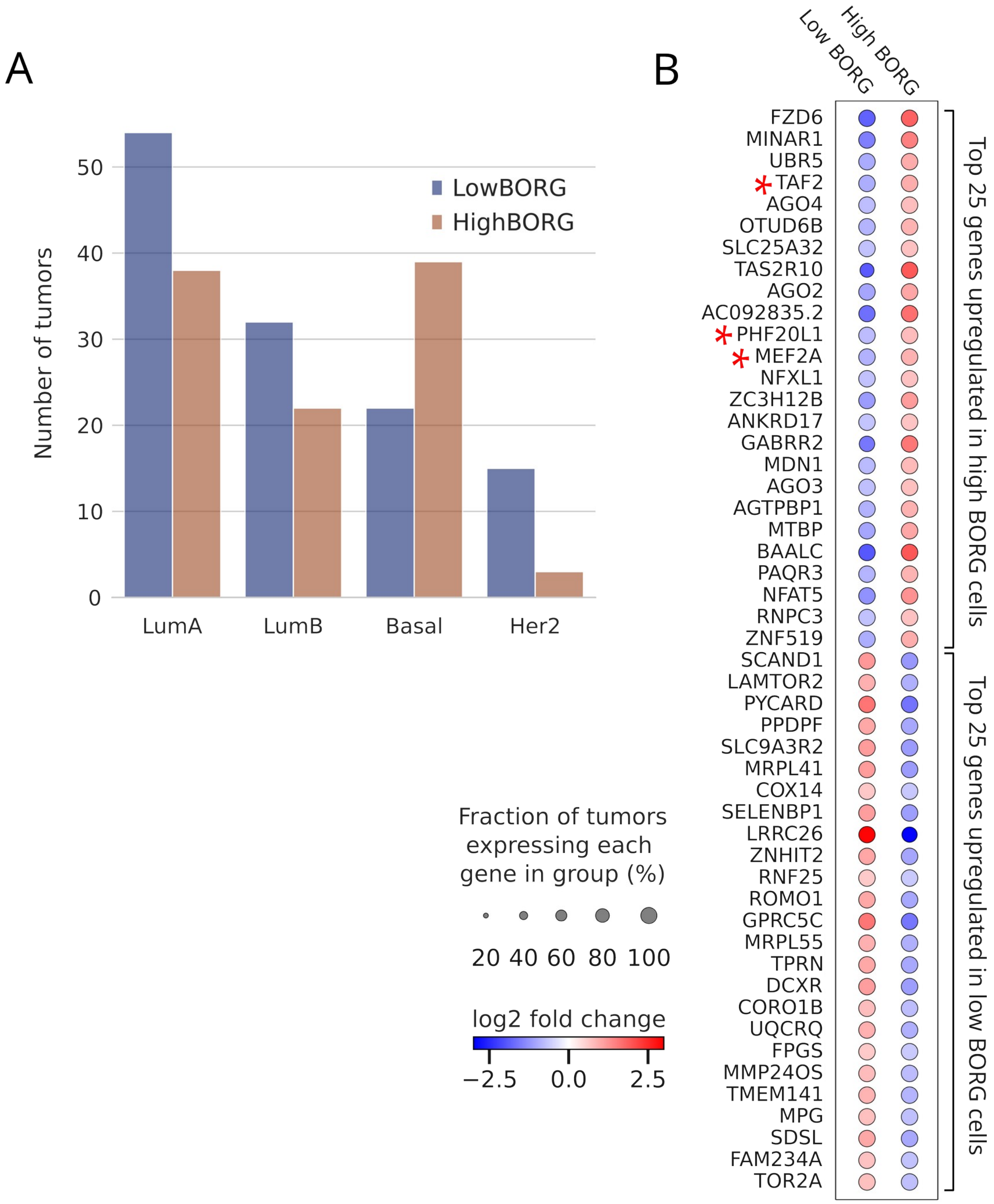

Fig. S5: Comparison of tumors with higher *versus* lower expression of BORG regardless of subtype. A. Number of tumors in the high and low BORG-expressing tumors in each PAM50 subtype of breast cancer tumors. All basal tumors included in this study are also triple negative and vice versa (see Methods). B. Several key cancer-related genes (asterisks) are among the top 25 genes upregulated in $BORG^{High}$ cells across all BC subtypes.

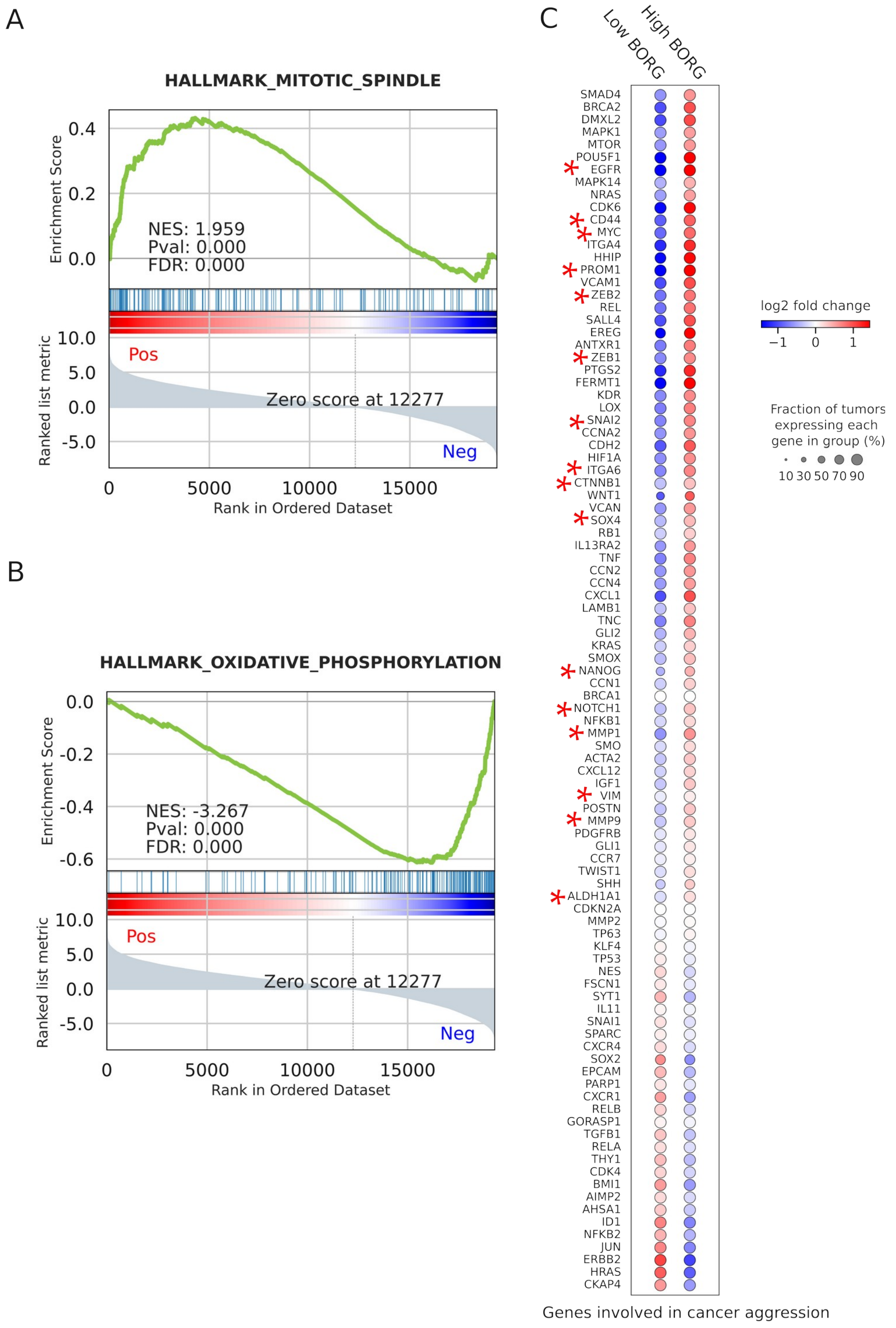

Fig. S6: Key cancer-related genes and pathways are differentially enriched in BORG^{High} *versus* BORG^{Low} tumors across all breast cancer subtypes. A and B: enrichment plots for the most positively- and negatively-enriched pathways in BORG^{High} tumors across all breast cancer subtypes. Vertical dark blue bars mark the position of genes differentially expressed in high *versus* low BORG tumors that are part of the indicated pathways. The color bar below the vertical bars identifies upregulated genes (shades of red) and downregulated genes (shades of blue), with the genes sorted based on differential expression value from left (most upregulated genes) to right (most downregulated genes). Genes marked by red are upregulated in BORG^{High} tumors compared to their BORG^{Low} counterparts. C.

Expression of genes known to be involved in induction of aggressive tumor behavior is increased in $BORG^{High}$ tumors. The expression pattern of all breast cancer aggression-related genes is shown including those that show mild or no changes. The genes in this list were selected from genes reported in the literature to be involved in breast cancer invasiveness. Asterisks mark genes known to be associated with basal or breast cancer stem cell phenotype.

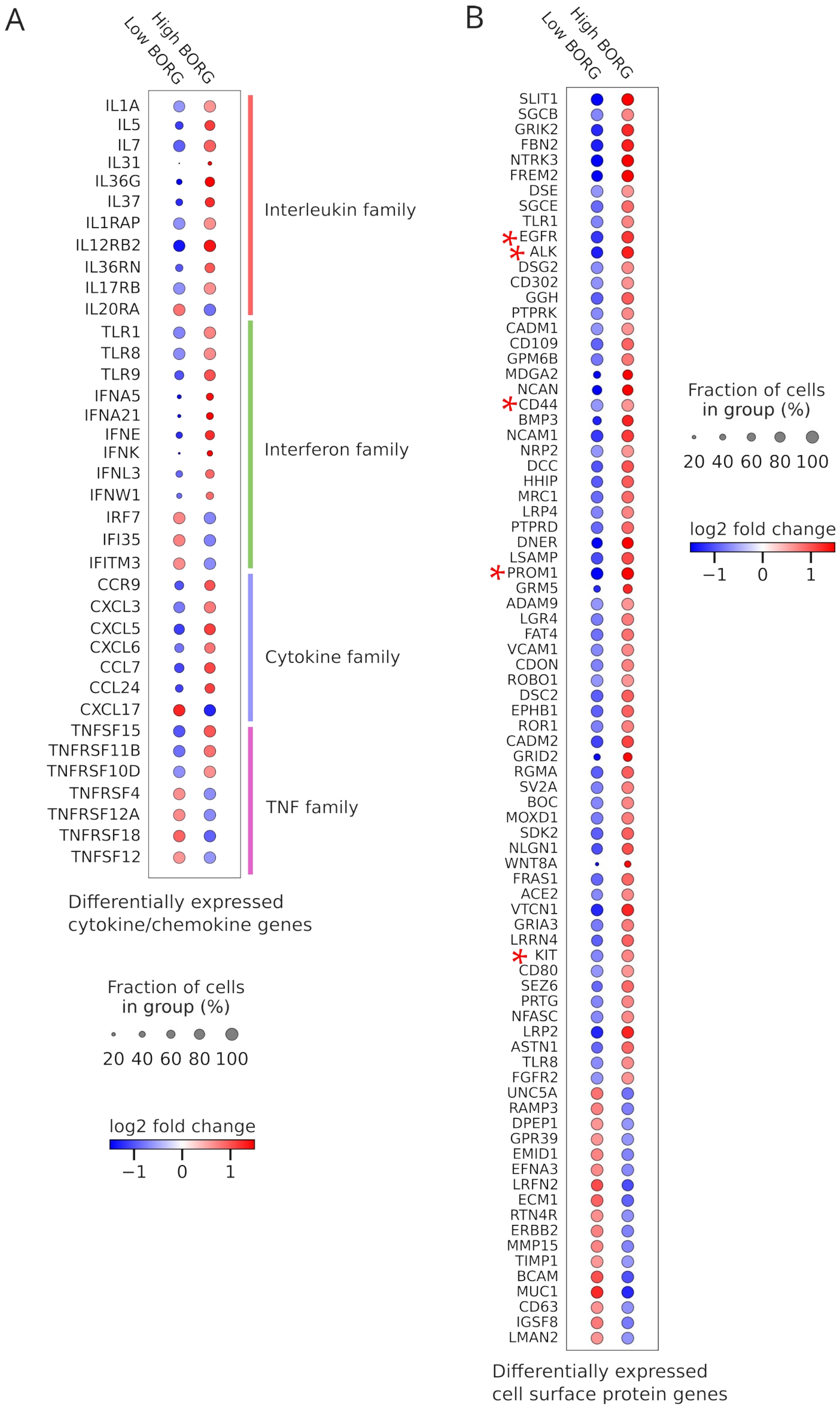

Fig. S7. BORG^{High} tumors upregulate multiple cytokines and cell surface proteins compared to BORG^{Low} tumors across all BC subtypes. A. Changes in the expression pattern of multiple cytokines and chemokines from the interferon (IFN), interleukin (IL), and tumor necrosis factor (TNF) families are shown in high and low BORG tumors. B. BORG^{High} tumors upregulate the expression of several cell surface proteins at mRNA level, including key breast cancer aggression factors (asterisks) compared to BORG^{Low} tumors.

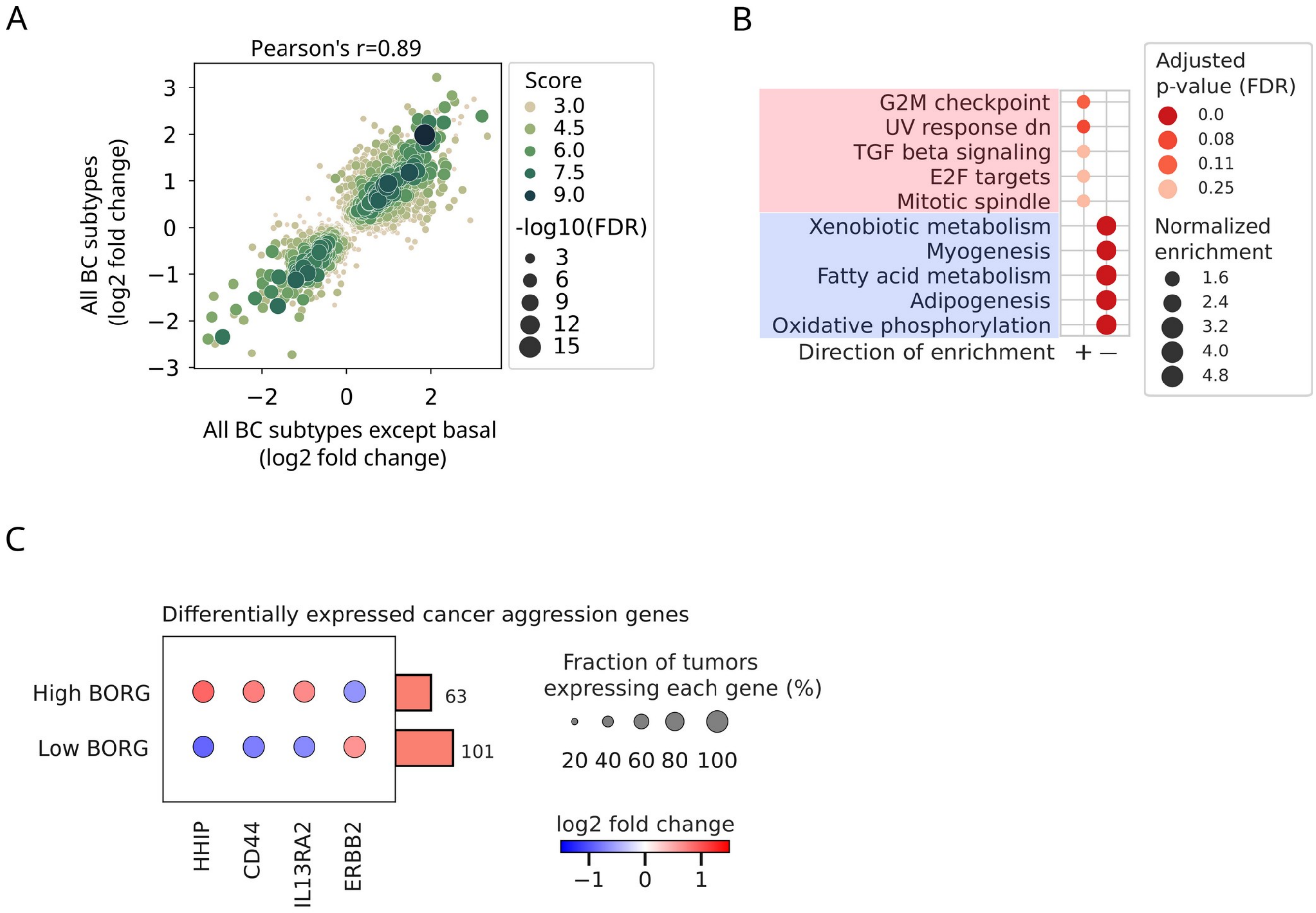

Fig. S8: The transcriptomic signature observed in BORG^{High} tumors across all breast cancer subtypes persists after the removal of the basal TNBC subset of tumors. A. Differential expression pattern of genes in BORG^{High} *versus* BORG^{Low} tumors were compared in all breast cancers irrespective of subtypes (y axis) or after eliminating basal, TNBC tumors from the comparison group (x axis). Each circle represents a single gene, with the size and hue of the circle determined by the FDR and the absolute value of z-score (labeled as “score” in the figure) values, respectively. The calculated Pearson correlation coefficient indicates that the two comparisons yield very similar gene expression patterns, thereby identifying a BORG-specific transcriptomic signature for high BORG expression independent of breast cancer subtype. B. Pathway analysis on genes differentially expressed in BORG^{High} *versus* BORG^{Low} non-basal, non-TNBC tumors is largely similar to the pattern observed when all breast tumor subtypes including basal TNBCs are used (Fig. 2A). C. Despite significant similarities, the number of genes involved in breast cancer aggression that are differentially expressed in high *versus* low BORG tumors is much smaller after removal of the basal TNBC tumors from the analysis.

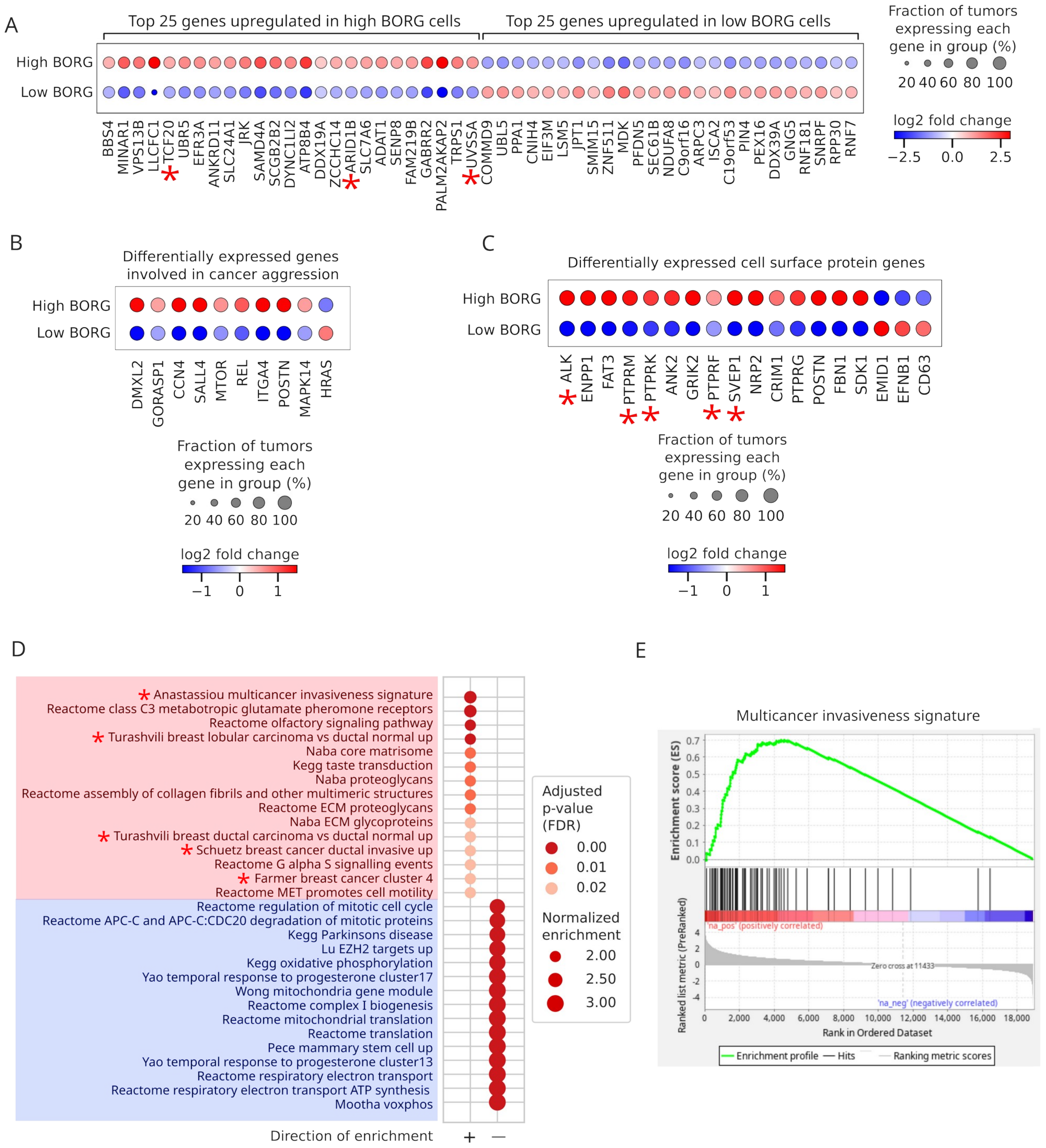

Fig. S9: High levels of BORG in TNBC tumors are associated with increased tumor invasiveness and pluripotency. A. Top differentially expressed genes in BORG^{High} *versus* BORG^{Low} tumors in the basal TNBCs. Key genes involved in pluripotency and induction of neoplastic process are marked with asterisks. B. Increased expression of multiple cancer invasiveness genes in BORG^{High} tumors. C. Several cell surface proteins are upregulated at mRNA level in BORG^{High} basal TNBC tumors, including those involved in key neoplastic processes (marked by asterisks). D. Enrichment analysis using the entire set of gene lists from the largest gene list database (C2) of mSigDB, which indicated that the positive enrichment of multiple breast cancer-related pathways and signatures (asterisks), including the multi-cancer invasiveness signature gene list. For the enrichment pattern of the subset of

gene lists related to cancer, please see Fig. 4B. E. Enrichment plot for the multi-cancer invasiveness signature gene list. Vertical black bars mark the position of genes differentially expressed in high *versus* low BORG basal TNBC tumors that are part of the multi-cancer invasiveness gene list. The color bar below the vertical bars identifies upregulated genes (shades of red) and downregulated genes (shades of blue), with the genes sorted based on differential expression value from left (most upregulated genes) to right (most downregulated genes). Genes marked by red are upregulated in $BORG^{High}$ basal TNBC tumors compared to $BORG^{Low}$ ones.

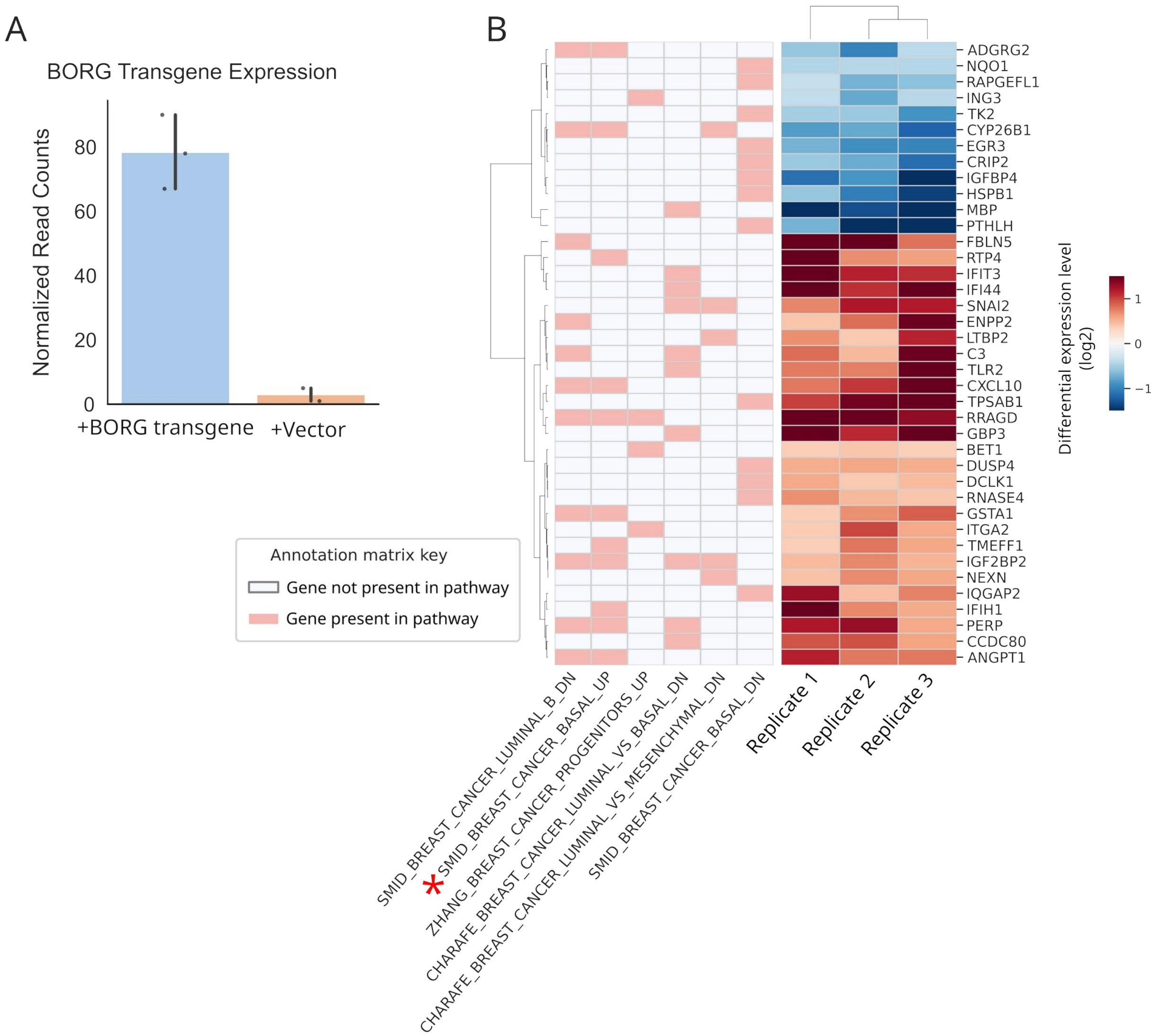

Fig. S10. BORG overexpression results in enrichment of genes specific to basal tumors and breast cancer progenitor cells. A. Forced mouse Borg overexpression from a transgene in D2OR cells leads to a strong rise in the cellular level of Borg as measured by RNA-seq. B. Top pathways enriched following Borg overexpression in D2OR cells include those specific to basal breast tumors (Smid breast cancer basal up) and breast cancer progenitors. Top 39 (out of 77) BORG induced genes that map to the 6 most enriched pathways are shown. The heatmap to the right shows the level of differential expression of each gene in three replicate studies. The annotation matrix to the left indicates the membership of each gene in the pathways named at the bottom. While genes expected to be upregulated in basal BC tumors and BC progenitors are indeed positively enriched after Borg overexpression, those expected to be downregulated in basal BC tumors are also negatively enriched.

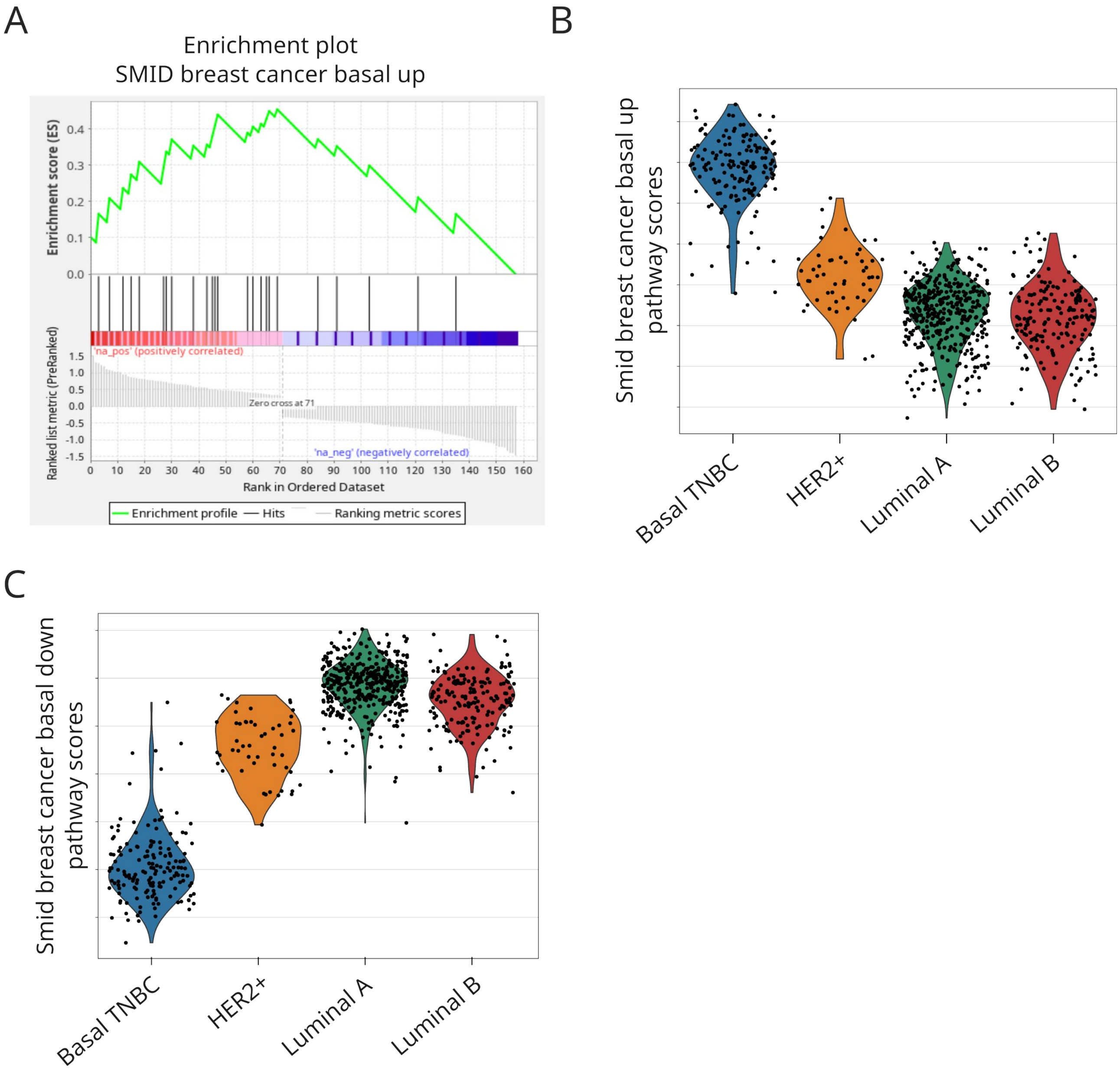

Fig. S11. Higher BORG expression levels induce a basal, aggressive phenotype observed in BORG^{High} TNBCs. A. Amongst the genes that are upregulated in both BORG-expressing D2.OR cells and BORG^{High} basal TNBC tumors, a significant fraction map to the Smid breast cancer basal up pathway. Vertical black bars mark the position of shared differentially expressed genes that are part of the 'Smid breast cancer up' pathway. The color bar below the vertical bars identifies upregulated genes (shades of red) and downregulated genes (shades of blue), with the genes sorted based on differential expression value from left (most upregulated genes) to right (most downregulated genes). Genes marked by red are the shared upregulated genes, while those in blue are the shared downregulated genes present in both BORG-expressing D2.OR cells (compared to vector-transfected control D2.ORs) and in BORG^{High} basal TNBC tumors (compared to low BORG ones). B. Validation of the upregulation of the 'Smid breast cancer basal up' pathway in basal TNBC cells compared to the other BC subtypes. Each dot corresponds to a tumor. The values shown are the enrichment scores for the pathway in each tumor. C. A similar validation study for the Smid breast cancer basal down gene list, indicating that the genes

included in these pathways are indeed up- (panel B) and down-regulated (panel C) in the basal TNBC tumors in the TCGA BRCA dataset.
